# Performance-based financing for improving HIV/AIDS service delivery: a systematic review

**DOI:** 10.1186/s12913-016-1962-9

**Published:** 2017-01-04

**Authors:** Amitabh B. Suthar, Jason M. Nagata, Sabin Nsanzimana, Till Bärnighausen, Eyerusalem K. Negussie, Meg C. Doherty

**Affiliations:** 1 Department of HIV/AIDS, World Health Organization, 20 Avenue Appia, CH-1211 Geneva 27, Switzerland; 2 Department of Pediatrics, Stanford University School of Medicine, Stanford, USA; 3 Rwanda Biomedical Center, Kigali, Rwanda; 4 Basel Institute for Clinical Epidemiology and Biostatistics, Swiss Tropical and Public Health Institute, Basel, Switzerland; 5 Institute of Public Health, Faculty of Medicine, Heidelberg University, Heidelberg, Germany; 6 Harvard T.H. Chan School of Public Health, Boston, USA; 7 Africa Health Research Institute (AHRI), Somkhele and Durban, South Africa

**Keywords:** Universal health coverage, Health financing, HIV, AIDS, Service, HIV testing, HIV treatment, Antiretroviral therapy, Quality, Access, Efficiency

## Abstract

**Background:**

Although domestic HIV/AIDS financing is increasing, international HIV/AIDS financing has plateaued. Providing incentives for the health system (i.e. performance-based financing [PBF]) may help countries achieve more with available resources. We systematically reviewed effects of PBF on HIV/AIDS service delivery to inform WHO guidelines.

**Methods:**

PubMed, WHO Index Medicus, conference databases, and clinical trial registries were searched in April 2015 for randomised trials, comparative contemporaneous studies, or time-series studies. Studies evaluating PBF in people with HIV were included when they reported service quality, access, or cost. Meta-analyses were not possible due to limited data. This study is registered with PROSPERO, number CRD42015023207.

**Results:**

Four studies, published from 2009 to 2015 and including 173,262 people, met the eligibility criteria. All studies were from Sub-Saharan Africa. PBF did not improve individual testing coverage (relative risk [RR], 1.00, 95% confidence interval [CI] 0.89 to 1.13), improved couples testing coverage (RR 1.11, 95% CI 1.02 to 1.20), and improved pregnant women testing coverage (RR 1.29, 95% CI 1.28-1.30). PBF improved coverage of antiretrovirals in pregnant women (RR 1.55, 95% CI 1.50 to 1.59), infants (RR 1.92, 95% CI 1.84 to 2.01), and adults (RR 1.74, 1.64 to 1.85). PBF reduced attrition (RR 0.84, 95% CI 0.74 to 0.96) and treatment failure (odds ratio 0.55, 95% CI 0.32 to 0.97). Potential harms were not reported.

**Conclusions:**

Although the limited data suggests PBF positively affected HIV service access and quality, critical health system and governance knowledge gaps remain. More research is needed to inform national policymaking.

**Electronic supplementary material:**

The online version of this article (doi:10.1186/s12913-016-1962-9) contains supplementary material, which is available to authorized users.

## Background

Political commitment, social mobilisation, and funding have increased rapidly since the 2001 global declaration to address HIV/AIDS [1]. This has translated into substantial progress in controlling the epidemic, including meeting the United Nations General Assembly target of expanding access to antiretroviral therapy (ART) to over 15 million people in 2015 [2]. This scale-up has helped avert 7.6 million deaths, thereby gaining 40.2 million life-years [3]. Scale-up of combination prevention, including ART, also contributed to the 30 million HIV infections averted since 2000 [2]. Although these achievements are impressive, HIV remains a leading cause of death and disability globally [4]. In 2014, there were an estimated 2.0 million new HIV infections, 1.2 million AIDS deaths, and 36.9 million people living with HIV [2]. Moreover, although domestic HIV/AIDS financing is increasing, international financing to control the epidemic has plateaued since 2011 [2]. Innovative health systems approaches may help countries achieve the new global target to end the HIV/AIDS epidemic by 2030 with the same resources or less [5].

Universal health coverage, i.e. ensuring all people obtain the health services they need without suffering financial hardship, has emerged as the health system foundation for the post-2015 global strategy for HIV, viral hepatitis, and sexually transmitted infections [6]. Essential elements of universal health coverage include health financing, essential medicines and health products, national health policies, health workforce, health statistics and information systems, and service delivery [7]. Health financing considers how funds are mobilised, pooled, and invested within health systems [8, 9]. For example, in some low- and middle-income countries, HIV/AIDS funds could be mobilised from tax revenue, social health insurance, the Global Fund (GFATM), and/or the United States President’s Emergency Plan for AIDS Relief (PEPFAR). These domestic and external funds are sometimes pooled within national programmes and then invested into services, medicines, and diagnostics needed for epidemic control. Traditionally, governments use input-based financing (IBF), wherein commodity, infrastructure, and human resource need is forecasted, to inform budgeting within national strategic plans. In recent years, financial incentives have been introduced in some countries to improve service delivery [10]. There is a wide range of terminology used for financial incentives [10]. Results-based financing broadly considers the use of incentives for clients or the health system [10]. Conditional cash transfers generally consider the use of financial incentives to clients for certain behaviours [11, 12]. For example, studies have provided conditional cash transfers for changes in sexual behaviour, receiving HIV testing, linking to care, and achieving viral suppression [13–15]. Performance-based financing (PBF) predominantly provides financial incentives for the health system based on the quantity and quality of health services [10].

PBF has been implemented in a number of countries with potential increases in service coverage, quality of care, and financial efficiency [16–21]. However, PBF could also skew focus and priorities to incentivised targets at the expense of other essential services [22]. Previous systematic reviews have evaluated PBF in a range of geographic contexts and disease programmes [23–25]. Since this time, evidence has emerged on PBF for HIV/AIDS service delivery within a health systems framework. We systematically reviewed this evidence to inform the development of the 2016 World Health Organization Consolidated Guidelines on the Use of Antiretroviral Drugs for Treating and Preventing HIV Infection [26].

## Methods

### Conduct

The systematic review was conducted in accordance with the Preferred Reporting Items for Systematic Review and Meta-Analysis (PRISMA) statement using a pre-defined protocol (International Prospective Register of Systematic Reviews [PROSPERO] identification number: CRD42015023207) [27, 28]. The PubMed and WHO Index Medicus databases were systematically searched without language, publication, date, or any other limits (Additional file 1: Text S1). Databases from the International AIDS Society, Conference on Retroviruses and Opportunistic Infections, and HIV/AIDS Implementers’ Meeting were also searched. The WHO International Clinical Trials Registry Platform, the Cochrane Central Register of Controlled Trials, the International Standard Randomised Controlled Trial Number Register, and ClinicalTrials.gov were searched for future and on-going studies. Experts in the field were contacted to identify unpublished research and on-going studies.

### Eligibility criteria

Per recommendations from the PRISMA group, eligibility criteria were based on key study characteristics: population, intervention, comparator, outcome, and design [27]. Specifically, studies were included when (1) the study population was composed of infants, children, adolescents, or adults with HIV in low- and middle-income countries; (2) the intervention was performance-based financing (i.e. any program that rewards the health system for delivery of one or more outputs or outcomes by one or more incentives, financial or otherwise, upon verification that the agreed-upon result has actually been delivered [10, 29]); (3) the comparator was no performance-based financing; (4) the outcomes were quality (including retention, viral suppression, adherence to national standard of care, patient satisfaction, patient centeredness), access (including HIV testing uptake and coverage, linkage to HIV care, treatment uptake, or treatment coverage), cost of HIV services, or harm; and (5) the study design was a randomised trial, comparative contemporaneous study, or time series study. Articles focusing on incentives for clients of HIV services were not included.

### Study screening and extraction

ABS and JMN independently screened abstracts of all identified articles and then matched the full texts of all articles selected during screening against the inclusion criteria. Studies meeting the inclusion criteria were included in the review and their references were searched for relevant studies. ABS and JMN completed the data extraction of eligible studies of study participants, methods, outcomes and quality assessment using a standardised spread-sheet. Disagreements in study screening and extraction were resolved by discussion.

### Statistical analyses

Relative risks were used to calculate effect sizes (Additional file 2: Text S2) [30]. When reported, study estimates from multivariable analyses were included. Given that no outcome was reported in more than one study in the same population, it was not possible to conduct meta-analyses.

### Quality assessment

For the quality assessment, studies were stratified based on study design (i.e. randomised controlled trial or observational study). Per recommendations from the Cochrane Collaboration, the Collaboration’s ‘Risk of bias’ tool was used to assess bias in randomised trials [31]. This tool rates studies based on four sources of bias: selection bias, performance and detection bias, attrition bias and reporting bias. Per recommendations from the Cochrane Collaboration [31], the *Newcastle-Ottawa Quality Assessment Scale* was adapted to assess bias in observational studies [32]. This scale rates studies based on criteria in three sources of bias: selection bias, confounding and measurement bias.

## Results

**Fig. 1:**
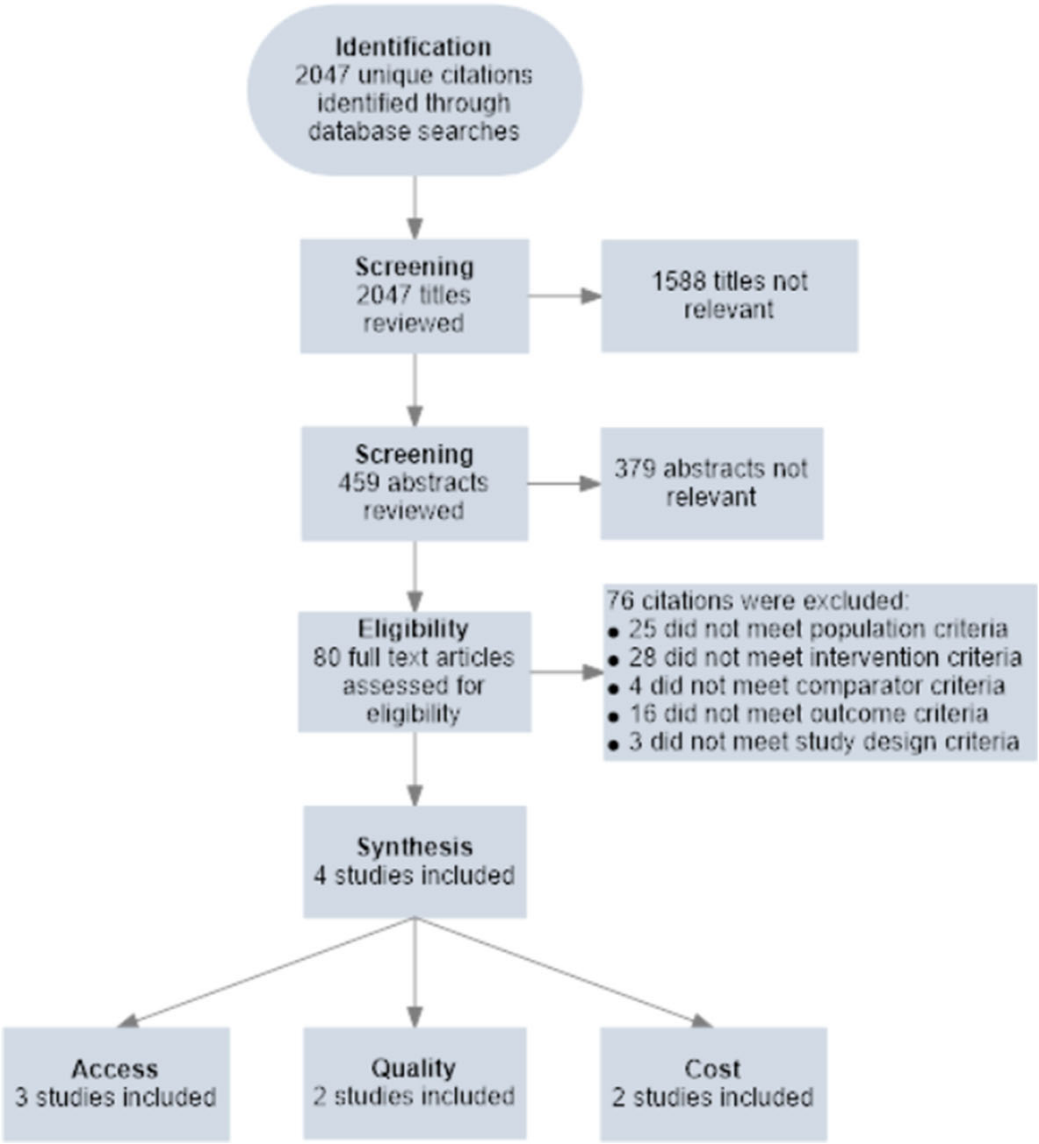
Study selection

2047 unique citations were identified through the databases searches. After screening all titles, 1588 citations were excluded. After screening the remaining 459 abstracts, 379 abstracts were excluded. 80 full texts were examined for eligibility, and 76 were excluded. Four studies, published from 2009 to 2015 and including 173,262 participants, met the eligibility criteria (Table and Fig. 1). One study was a cluster randomised trial [33] while the other three articles were observational studies [34–36]. Two studies were from Cote d’Ivoire, one study was from Rwanda, and one study was from Kenya (Table 1). The clinical trial registers identified two on-going trials: one in Zambia and one in Zimbabwe [37, 38].

**Table 1:** Study methods

Author (Year)	Study Setting	Study Design	Years	Follow-up	Intervention group	Comparator group	Outcomes	Analytic Model	Losses to follow-up
Attiah (2010) [34]	116 health facilities in Cote d'Ivoire^a^	Contemporaneous observational study	2008-2009	18 months	Facilities implementing PBF	Different facilities not implementing PBF	-% of women that received HTC -% of pregnant women that received ARV prophylaxis -% of infants that received ARV prophylaxis	Crude risk ratios	Not relevant (HIV testing and ARV prophylaxis reported)
DeWalque (2015) [33]	24 health facilities in Rwanda	Cluster randomised trial	2006-2008	14-18 months	Facilities implementing PBF	Different facilities not implementing PBF	-% of all individuals receiving HIV testing -% of couples receiving HIV testing	Risk ratios adjusted for year, age, gender, years of schooling, and household wealth	Not relevant (HIV testing reported)
Odeny (2013) [35]	60 health facilities in Kenya	Contemporaneous observational study	2007-2012	6-12 months	Facilities implementing PBF	Different facilities not implementing PBF	-Treatment failure (CD4 persistently below 100 cells/mm3 after 6–12 months of ART, CD4 falls by ≥50% from on treatment peak value, CD4 falls to or below pre-ART level)	Odds ratio adjusted baseline patient characteristics, year of ART initiation, and CD4 cell count at initiation	Not reported
Tanoh (2009) [36]	4 health facilities in Cote d'Ivoire^a^	Time-series observational study	2005-2007	24 months	Facilities implementing PBF	Same facilities before they implemented PBF	-ART coverage -Attrition after 12 months	Crude risk ratios	Not reported

^a^Both studies were from the same PBF initiative

**Fig. 2:**
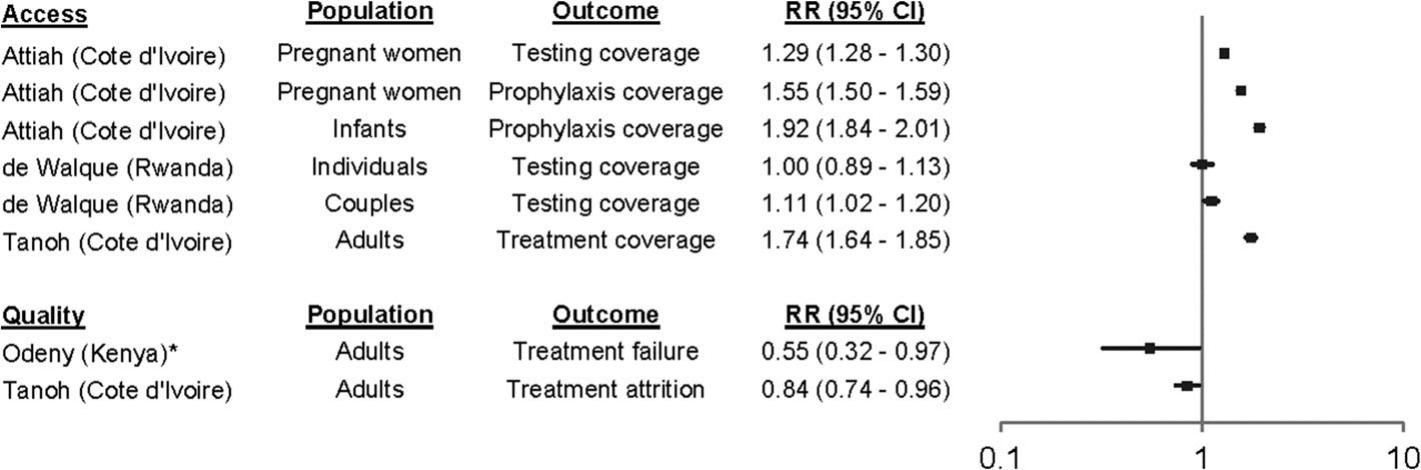
Effects of PBF on HIV service access and quality. *Adjusted odds ratio rather than relative risk presented. Treatment failure defined as CD4 persistently below 100 cells/mm3 after 6–12 months of ART, CD4 falls by ≥50% from on treatment peak value, and/or CD4 falls to or below pre-ART level

Three studies reported on access to HIV services (Fig. 2) [33, 34, 36]. One study was randomised and the others were observational (Table 1). Two studies were in Cote d’Ivoire while the other was in Rwanda (Table 1). The average duration of studies was 1.6 years (Table 1). The cluster randomised trial did not report sequence generation and allocation concealment [33] (Additional file 3: Table S1). Two of the observational studies did not control for confounding [34, 36] and one did not report losses to follow-up [27] (Additional file 4: Table S2). Although there was no change in individual testing coverage (relative risk [RR], 1.00, 95% confidence interval [CI] 0.89 to 1.13), there was an improvement in couples testing coverage (RR 1.11, 95% CI 1.02 to 1.20) [33]. There was also an observed increase in pregnant women testing coverage (RR 1.29, 95% CI 1.28-1.30) [34]. There were observed improvements in coverage of antiretrovirals for preventing mother-to-child transmission in pregnant women (RR 1.55, 95% CI 1.50 to 1.59) and infants (RR 1.92, 95% CI 1.84 to 2.01) [37]. There was also an observed improvement in adult ART coverage (RR 1.74, 1.64 to 1.85) [36].

Two studies reported on quality of HIV services (Fig. 2) [35, 36]. Both studies were observational (Table 1). One study was in Cote d’Ivoire while the other was in Rwanda (Table 1). The average duration of the studies was 1.4 years (Table 1). Neither of the studies reported losses to follow-up and one did not adjust for confounding (Additional file 4: Table S2). PBF was associated with a reduction in patient attrition (RR 0.84, 95% CI 0.74 to 0.96) [36] and a reduction in rates of treatment failure (odds ratio 0.55, 95% CI 0.32 to 0.97) [35].

Three studies described their performance-based financing schemes (Table 2) [33, 34, 36]. PBF was provided for HIV testing, adult treatment and care, paediatric treatment and care, and prevention of mother-to-child transmission. Paediatric treatment and care was incentivised at rates higher than other populations. One study described how the scheme was monitored; health facilities were required to submit monthly reports and quarterly requests for payment to district committees responsible for verifying the quality of the data and authorising payment [33]. The committees verified that the data reported were the same as what was recorded in facility records during unannounced quarterly visits. There was no description of whether independent third parties were involved in verification of data. The cost of the monitoring component of PBF was not reported in any of the studies. Estimates were not available on the overall comparative cost of PBF to IBF.

**Table 2:** Performance-based financing service indicators and unit payments [33, 34, 36]

Service	Rwanda (2006 US$)	Côte d’Ivoire (2007 US$)
HIV testing and counselling		
Number of clients tested for HIV	0.92	3
Number of couples/partners tested	4.59	7.5
Number of children of HIV+ parents tested	-	7.5
Adult treatment and care		
Number of HIV+ patients who received CD4 test	4.59	-
Number of new HIV+ adults on ART	4.59	7.5
Number of HIV+ patients on co-trimoxazole prophylaxis	0.46	-
Number of HIV+ patients screened for tuberculosis	2.75	-
Number of facility visits (eligibility, 15 days, 1 month, 3 months, 9 months, 12 months, unplanned)	-	3-7.5
Number of facility visits for ART-ineligible patients (quarterly)	-	6
Paediatric treatment and care		
Number of new children living with HIV on ART	-	11.25
Number of facility visits (eligibility, 15 days, 1 month, 3 months, 9 months, 12 months, unplanned)	-	4.5-11.25
Number of facility visits for ART-ineligible patients (quarterly)	-	9
Prevention of mother-to-child transmission		
Births at health facility	-	6
Number Postnatal visits	-	3
Number of infants born to HIV+ mothers tested	9.17	-
Number of HIV+ women on contraception	2.75	-
Number of HIV+ pregnant women on ART during labour	4.59	-
Number of new HIV-infected infants on ART	6.88	-

The US$ figures represent an individual service unit

## Discussion

Cumulatively, the body of evidence suggested that PBF could have a role for the prevention of mother-to-child transmission cascade by improving coverage of testing in pregnant women, couples testing, and antiretrovirals in the pregnant women and their infants. There was also potential for PBF to improve ART coverage, retention, and adherence in adults. However, many health system and governance knowledge gaps were identified. For example, there were no data on potential harms associated with PBF or on spill-over effects on overall health system performance. Also, all data were generated in generalised epidemics. We have formulated the critical knowledge gaps identified into research priorities (Table 3).

**Table 3:** Possible performance-based financing research priorities

Category	Research priority
Service delivery	1) Evaluate effect size over longer periods of time
	2) Evaluate effects on non-incentivised services and the broader health system
	3) Evaluate effects of stopping PBF
	4) Identify specific services for which PBF is effective
	5) Validate results in different geographic settings
Human resources	6) Quantify and compare human resources required for implementing and monitoring PBF and IBF
	7) Evaluate effects on health workforce behaviour, including absenteeism, task sharing, and productivity
Governance	8) Evaluate effectiveness of PBF as vertical approach within the HIV/AIDS programme versus as part of overall health system
	9) Identify potential health system mechanisms through which PBF may be effective
Health financing	10) Quantify and compare overall cost, including schemes and oversight, of PBF and IBF
Information systems	11) Evaluate effects on the quality, accuracy, completeness, timeliness, and use of data at health facilities
	12) Evaluate the role of communities and PLHIV organisations in validating performance
Clients	13) Evaluate and compare acceptability of PBF and IBF services

PBF was associated with improved quality of care by reducing rates of attrition and treatment failure. Since some interventions have shown promise in reducing rates of treatment failure in individual studies but did not replicate their results in other studies, the PBF results require further examination and validation in other geographic contexts [39]. Risk factors for patient attrition have been related to access, acceptability, and affordability of services [26]. Studies evaluating interventions to improve access and affordability have consistently shown favourable outcomes [40]. It is possible that PBF’s positive effects on attrition are related to making provider behaviour more acceptable to clients and thereby improving patient satisfaction and utilisation of HIV services. This means PBF could complement interventions that improve access and affordability of HIV services [41].

PBF was shown to improve access to HIV services. This is consistent with results from other disease programmes [16, 18, 20]. However, if health workforce remuneration is based primarily on specific indicators selected for HIV PBF, it is possible that other health indicators not based on performance may get de-prioritised. This could lead to health sector inequities wherein incentivised HIV services are given increased attention relative to non-incentivised services for HIV and other diseases. To prevent this, some publicly financed PBF initiatives include indicators for many diseases [20]. This approach would make PBF a wider health financing reform and may lead to improved institutionalisation and larger returns on health investments [42]. Consideration could also be made to evaluate use of PBF across different ministries as overall government reform.

The evidence on costing was limited to the PBF schemes used in studies. It is difficult to directly compare the PBF schemes used in studies due to potential economic and health system differences across countries. For example, it is possible that facilities in Cote d’Ivoire had larger incentives than facilities in Rwanda for the same indicators due to differences in health workforce remuneration or national guideline laboratory reagent requirements. Nonetheless, the funds allocated per indicator seemed to depend on a number of criteria including priority within the health sector, complexity of the task, and the required health commodities and staff time [43]. Moreover, although the PBF schemes described the service being incentivised, there was no description of other operational costs associated with implementing PBF. For example, if personnel are required to oversee the scheme and ensure quality data are being reported for reimbursement, this may require additional financial and human resources [44]. Before national implementation, it would be necessary to understand the overall resource requirements of PBF to IBF. If PBF requires substantially more resources than IBF, other potentially more cost-effective and sustainable options to improve service access and quality could be explored.

The identified studies used external grants for PBF and were relatively short in duration. External funds may not be subject to the same regulations and restrictions as domestic funds. For example, using PBF for domestic funds likely has regulatory and procedural implications that extend beyond the Ministry of Health [45]. In order to ensure sustainability of PBF within HIV/AIDS programmes, issues surrounding the transferring of domestic funds should be reviewed prior to implementing PBF. The short duration of studies also mean that it is unknown whether the effects wane or improve over time. For example, workforce behaviour change could wane over time as they eventually expected the bonus to be a part of their normal salary. Moreover, no studies evaluated the effects of withdrawing PBF schemes on service quality and access. This is a research priority as it is possible that stopping PBF schemes could cause previously incentivised services to have levels of quality or access that were even lower than baseline due to newly found expectations from health system staff.

Implementing PBF requires a number of health system actions [10]. First, indicators to include the PBF scheme must be identified and agreed upon. The selection of indicators to be incentivised should be based on pre-agreed criteria, including being patient-centred, to prevent potential harm [46]. Selection of indicators that do not always benefit the patient could result in undesired health system behaviour. For example, in relation to preventing mother-to-child transmission of HIV, if the number of caesarean sections is reimbursed based on performance, it is possible that providers could provide caesarean sections to women who do not need them. Second, facilities must further develop their budget management and administration skills. For example, currently many districts have to report quantities of commodities that they have forecasted in order to receive central government funds. With PBF, facilities would have to become more autonomous in a sense by managing and disbursing the funds for health staff, commodities, and infrastructure at the local level. Third, in order to prevent undesired behaviour, an oversight system must be put in place to ensure the robustness and quality of the PBF data and services being reported.

PBF’s effects on health information systems were not reported in any of the studies. Often, lower tiers of the health system collect, analyse, and interpret data mostly for reporting purposes [47]. With PBF, health staff are incentivised to carefully collect, analyse, and interpret facility-level data. This incentive may help develop information system capacities within the workforce. This capacity development may lead health staff to understand, interpret and act upon other routinely collected programme data (including those for non-incentivised services). Therefore, PBF could improve completeness, accuracy, timeliness, and use of health data. This could also improve procurement and supply chain management systems by improving the capacity of health facilities to forecast commodity needs. These potential spill-over effects on the health system require further examination.

The studies did not fully describe the mechanisms through which PBF may have been effective. More research could explain how PBF affects staff behaviour. For example, it was hypothesised that PBF works primarily by motivating existing healthcare staff to work harder and more efficiently and thereby improve productivity; however, some settings may already have healthcare staff that are working their hardest and as efficiently as their facility allows [48]. It was also hypothesised that PBF could make health workforce salaries more competitive, but some settings may already compensate health workforce with competitive salaries [49]. In these situations, PBF may not provide additional benefits. Moreover, many healthcare staff may have been intrinsically motivated to provide care to members of their community. It is unknown whether PBF changes their motivation to be more extrinsic. Therefore, thorough baseline situation analyses of health workforce productivity and remuneration would help countries in considering possible benefits and harms of implementing PBF [50].

The studies did not describe secondary effects of PBF. For example, PBF may be a mechanism to increase accountability for PBF services across different tiers of the health system. This relates closely to the performance contracts that are used within PBF schemes. Although not reported in the identified studies, some national or local governments assign minimum levels of services that must be provided by health facilities. Remuneration in PBF schemes may only start after this minimum level of services has been provided. For example, health facilities may be assigned to test a minimum of 20 people for HIV every month. If this minimum threshold is not met, the health facility is not eligible for PBF because it has failed to achieve its assigned duties. Therefore, PBF could help ensure providers are accountable for providing the minimum level of services assigned by the government.

This systematic review identified studies evaluating the effects of PBF on the quality, access, and cost of HIV service delivery. It is possible that non-financial incentives, i.e. gifts, could affect healthcare staff behaviour in a more cost-effective manner [51]. Gifts could also prevent the development of hierarchical relationships between funders and recipients and undesired effects on intrinsic staff motivation. Given our focus on performance-based financing, we did not include articles including client-based incentives in this systematic review due to potential differences in health system implementation, financial resource requirements, and impact. We encourage thorough evaluation and publication of results-based financing initiatives that include incentives for both the health system and clients to inform policy and practice [52].

There are some methodological limitations that need to be considered when evaluating this evidence-base. One of the main limitations of this body of literature was that it was primarily observational in nature. Observational data are susceptible to confounders which could affect our confidence in the effect size. For example, the positive effects of PBF observed in the studies may have been due to more experienced health workforce in PBF facilities, higher salaries at PBF facilities, or more funds for HIV services at PBF facilities. In particular, the results of uncontrolled before-after studies should be interpreted with caution due to difficulties in attributing causality to PBF with this study design [53]. There were also concerns related to allocation concealment and sequence generation with the cluster-randomised trial. Finally, design of PBF evaluations requires careful planning and discussion among relevant stakeholders. During this systematic review several PBF evaluations were identified that were excluded because events without a denominator were reported. For example, rather than reporting coverage, the number of units of service administered in PBF facilities compared to non-PBF facilities were reported in some studies [54, 55]. Since the number of people eligible for these services may be higher in PBF facilities versus non-PBF facilities, or vice versa, it is difficult to draw any conclusion from these data. Finally, the scope of this review was limited to services covered in the 2016 World Health Organization Consolidated Guidelines on the Use of Antiretroviral Drugs for Treating and Preventing HIV Infection [26]; further research and synthesis is needed to evaluate the effects of PBF on the effectiveness of HIV counselling and education.

## Conclusion

In conclusion, the limited data available suggest possible benefits of PBF for expanding access to quality HIV services; however, critical health system and governance knowledge gaps remain. Moreover, the long term sustainability of PBF in the absence of external funds is unknown. Data filling these gaps are needed to inform national policy makers.

## Additional files

Additional file 1: Text S1. Database search strategies. (DOCX 12 kb)
Additional file 2: Text S2. Effect estimate calculations. (DOCX 23 kb)
Additional file 3: Table S1. Bias assessment of randomised trials. (DOCX 12 kb)
Additional file 4: Table S2. Bias assessment of observational studies. (DOCX 12 kb)

## Abbreviations

AIDS: Acquired immune deficiency syndrome
ART: Antiretroviral therapy
CI: Confidence interval
GFATM: The global fund to fight AIDS, tuberculosis
HIV: Human immunodeficiency virus
IBF: Input-based financing
PBF: Performance-based financing
PEPFAR: President’s emergency plan for AIDS relief
PRISMA: Preferred reporting items for systematic reviews and meta-analyses
PROSPERO: International Prospective Register of Systematic Reviews
RR: Relative risk
WHO: World Health Organization

## References

[1] The United Nations. Declaration of Commitment on HIV/AIDS. 2001. Available from: http://www.un.org/ga/aids/docs/aress262.pdf. [cited 24 June 2015].

[2] Joint United Nations Programme on HIV and AIDS. MDG6: 15 years, 15 lessons of hope from the AIDS response. 2015. Available from: http://www.unaids.org/sites/default/files/media_asset/MDG6Report_en.pdf. [cited 24 July 2015].

[3] World Health Organization. Global update on the health sector response to HIV, 2014. 2014. Available from: http://www.who.int/iris/bitstream/10665/128494/1/9789241507585_eng.pdf. [cited 4 June 2015].

[4] GBD 2013 Mortality and Causes of Death Collaborators (2015). Global, regional, and national age-sex specific all-cause and cause-specific mortality for 240 causes of death, 1990–2013: a systematic analysis for the Global Burden of Disease Study 2013. Lancet.

[5] United Nations General Assembly Resolution 70/1. Transforming our World: the 2030 Agenda for Sustainable Development. 2015. Available from: http://www.un.org/ga/search/view_doc.asp?symbol=A/RES/70/1&Lang=E. [cited 4 November 2015].

[6] World Health Organization. Global Health Sector Strategies for HIV / Viral Hepatitis / Sexually Transmitted Infections, 2016–2021. 2015. Available from: http://apps.who.int/iris/bitstream/10665/246178/1/WHO-HIV-2016.05-eng.pdf. [cited 20 June 2015].

[7] World Health Organization. Universal health coverage. 2014. Available from: http://www.who.int/mediacentre/factsheets/fs395/en/. [cited 9 June 2015].

[8] World Health Organization (2010). Health systems financing: the path to universal coverage.

[9] Roberts M, Hsiao W, Berman P, Reich M. Getting health reform right: a guide to improving performance and equity. New York City: Oxford University Press; 2008.

[10] The World Bank. Performance-Based Financing Toolkit. 2014. Available from: https://openknowledge.worldbank.org/bitstream/handle/10986/17194/9781464801280.pdf?sequence=1. [cited 25 June 2015].

[11] Lagarde M, Haines A, Palmer N (2007). Conditional cash transfers for improving uptake of health interventions in low- and middle-income countries: a systematic review. JAMA.

[12] Gopalan SS, Mutasa R, Friedman J, Das A (2014). Health sector demand-side financial incentives in low- and middle-income countries: a systematic review on demand- and supply-side effects. Soc Sci Med.

[13] Baird SJ, Garfein RS, McIntosh CT, Ozler B (2012). Effect of a cash transfer programme for schooling on prevalence of HIV and herpes simplex type 2 in Malawi: a cluster randomised trial. Lancet.

[14] Thornton RL (2008). The demand for, and impact of, learning HIV status. Am Econ Rev.

[15] El-Sadr W, Branson B, Hall HI, Beauchamp G, Donnell D, Torian L (2015). Effect of Financial Incentives on Linkage to Care and Viral Suppression: HPTN 065.

[16] Eichler R, Auxila P, Pollock J (2001). Output-based health care: paying for performance in Haiti. Public Policy Priv Sect.

[17] Hecht R, Batson A, Brenzel L (2004). Making health care accountable. Finance Dev.

[18] Soeters R, Griffiths F (2003). Improving government health services through contract management: a case from Cambodia. Health Policy Plan.

[19] Low-Beer D, Afkhami H, Komatsu R, Banati P, Sempala M, Katz I (2007). Making performance-based funding work for health. PLoS Med.

[20] Basinga P, Gertler PJ, Binagwaho A, Soucat AL, Sturdy J, Vermeersch CM (2011). Effect on maternal and child health services in Rwanda of payment to primary health-care providers for performance: an impact evaluation. Lancet.

[21] World Health Organization. Performance-based grants for reproductive health in the Philippines. 2011. Available from: http://whqlibdoc.who.int/hq/2011/WHO_RHR_11.04_eng.pdf. [cited 4 June 2015].

[22] Renmans D, Holvoet N, Orach CG, Criel B (2016). Opening the ‘black box’ of performance-based financing in low- and lower middle-income countries: a review of the literature. Health Policy Plan.

[23] Eldridge C, Palmer N (2009). Performance-based payment: some reflections on the discourse, evidence and unanswered questions. Health Policy Plan.

[24] Oxman AD, Fretheim A (2009). Can paying for results help to achieve the Millennium Development Goals? Overview of the effectiveness of results-based financing. J Evid Based Med.

[25] Houle SK, McAlister FA, Jackevicius CA, Chuck AW, Tsuyuki RT (2012). Does performance-based remuneration for individual health care practitioners affect patient care?: a systematic review. Ann Intern Med.

[26] World Health Organization. Consolidated guidelines on the use of antiretroviral drugs for treating and preventing HIV infection: Recommendations for a public health approach. 2016. Available from: http://apps.who.int/iris/bitstream/10665/208825/1/9789241549684_eng.pdf. [cited 29 June 2016]. 27466667

[27] Liberati A, Altman DG, Tetzlaff J, Mulrow C, Gotzsche PC, Ioannidis JP (2009). The PRISMA statement for reporting systematic reviews and meta-analyses of studies that evaluate health care interventions: explanation and elaboration. PLoS Med.

[28] Suthar AB. Role of performance-based financing for HIV/AIDS control: a systematic review and meta-analysis. 2015. Available from: http://www.crd.york.ac.uk/PROSPEROFILES/23207_PROTOCOL_20150504.pdf. [cited 9 June 2015].

[29] World Health Organization. Technical update on HIV incidence assays for surveillance and monitoring purposes. 2015. Available from: http://www.unaids.org/sites/default/files/media_asset/HIVincidenceassayssurveillancemonitoring_en.pdf. [cited 11 April 2015].

[30] Rothman K, Greenland S, Lash T. Modern epidemiology: third edition. Philadelphia: Lippincott Williams & Wilkins; 2008.

[31] The Cochrane Collaboration. Cochrane Handbook for Systematic Reviews of Interventions. Available from: http://www.cochrane-handbook.org/. [cited 19 April 2011].

[32] Wells G, Shea B, O'Connell D, Peterson J, Welch V, Losos M, et al. The Newcastle-Ottawa Scale (NOS) for assessing the quality of nonrandomised studies in meta-analyses. Available from: http://www.ohri.ca/programs/clinical_epidemiology/oxford.asp. [cited 23 February 2011].

[33] de Walque D, Gertler PJ, Bautista-Arredondo S, Kwan A, Vermeersch C, de Dieu BJ (2015). Using provider performance incentives to increase HIV testing and counseling services in Rwanda. J Health Econ.

[34] Attiah J, Ntumbanzondo M, Ghanotakis E, Katuala G, Pitter C, Buono N (2010). Performance-based financing: a mechanism to improve uptake pediatric and maternal HIV care and treatment.

[35] Odeny T, Gasasira A, Haakenstad A, Moore K, DeCenso B, Masters S (2013). Facility and patient-level determinants of treatment failure among Kenyan adults initiating antiretroviral therapy between 2007 and 2012.

[36] Tanoh AR, Attiah J, Fayama M, Essombo J, Guebo A (2009). Performance Based Financing: Evaluation of Programatic Results after two years of Implementation in Côte d’Ivoire.

[37] The World Bank. Impact evaluation of Zambia’s health results-based financing project. 2014. Available from: http://apps.who.int/trialsearch/Trial2.aspx?TrialID=ISRCTN14332616. [cited 20 June 2015].

[38] The World Bank. Impact evaluation of Zimbabwe’s health results-based financing project. 2014. Available from: http://apps.who.int/trialsearch/Trial2.aspx?TrialID=ISRCTN16392613. [cited 20 June 2015].

[39] Chaiyachati KH, Ogbuoji O, Price M, Suthar AB, Negussie EK, Barnighausen T (2014). Interventions to improve adherence to antiretroviral therapy: a rapid systematic review. AIDS.

[40] Kranzer K, Govindasamy D, Ford N, Johnston V, Lawn SD (2012). Quantifying and addressing losses along the continuum of care for people living with HIV infection in sub-Saharan Africa: a systematic review. J Int AIDS Soc.

[41] Suthar AB, Rutherford GW, Horvath T, Doherty MC, Negussie EK (2014). Improving antiretroviral therapy scale-up and effectiveness through service integration and decentralization. AIDS.

[42] Witter S, Toonen J, Meessen B, Kagubare J, Fritsche G, Vaughan K (2013). Performance-based financing as a health system reform: mapping the key dimensions for monitoring and evaluation. BMC Health Serv Res.

[43] The World Bank. Public ends, private means: strategic purchasing of health services. 2007. Available from: https://openknowledge.worldbank.org/bitstream/handle/10986/6683/399790PAPER0Pu101OFFICIAL0USE0ONLY1.pdf. [cited 8 July 2015].

[44] SINA Health. Performance-based financing in action: theory and instruments. 2015. Available from: http://www.sina-health.com/?page_id=585. [cited 8 July 2015].

[45] Meessen B, Soucat A, Sekabaraga C (2011). Performance-based financing: just a donor fad or a catalyst towards comprehensive health-care reform?. Bull World Health Organ.

[46] Baltussen R, Niessen L (2006). Priority setting of health interventions: the need for multi-criteria decision analysis. Cost Eff Resour Alloc.

[47] AbouZahr C, Boerma T (2005). Health information systems: the foundations of public health. Bull World Health Organ.

[48] World Health Organization. Workload Indicators of Staffing Need (WISN). 2010. Available from: http://whqlibdoc.who.int/publications/2010/9789241500197_users_eng.pdf. [cited 8 July 2015].

[49] The International Labour Organisation. The global wage report 2014/15: wages and income inequality. 2015. Available from: http://www.ilo.org/wcmsp5/groups/public/---dgreports/---dcomm/---publ/documents/publication/wcms_324678.pdf. [cited 8 July 2015].

[50] The World Health Organization. A guide to rapid assessment of human resources for health. 2004. Available from: http://www.who.int/hrh/tools/en/Rapid_Assessment_guide.pdf. [cited 8 July 2015].

[51] Van Lerberghe W, Conceicao C, Van Damme W, Ferrinho P (2002). When staff is underpaid: dealing with the individual coping strategies of health personnel. Bull World Health Organ.

[52] Health Results Innovation Trust Fund. Results-based financing projects in the health sector. 2015. Available from: http://rbfhealth.org/projects. [cited 1 November 2015].

[53] Cochrane Effective Practice and Organisation of Care Group. What study designs should be included in an EPOC review and what should they be called? 2015. Available from: http://epoc.cochrane.org/epoc-specific-resources-review-authors. [cited 1 November 2015].

[54] Shillingi L, Addison-Fynn M, Limage J, Amartey P, Ataarem P, Clemmons L. Routine monitoring, feedback and dialogue: key ingredients in performance-based funding and civil society capacity-building. Windhoek: The 2009 HIV/AIDS Implementers’ Meeting; 2009; 10–14.

[55] Zeng W, Rwiyereka AK, Amico PR, Avila-Figueroa C, Shepard DS (2014). Efficiency of HIV/AIDS health centers and effect of community-based health insurance and performance-based financing on HIV/AIDS service delivery in Rwanda. Am J Trop Med Hyg.

