# Supplementary material for: Performance-based financing for improving HIV/AIDS service delivery: a systematic review
Source: BMC Health Serv Res. 2017 Jan 4;17:6. doi: 10.1186/s12913-016-1962-9 (PMC5210258; doi:10.1186/s12913-016-1962-9)
Supplement: Additional file 1: — Text S1. Database search strategies. (DOCX 12 kb) [file 12913_2016_1962_MOESM1_ESM.docx]

**Text S1.** Database search strategies

Search strategy for MEDLINE

(HIV Infections[MeSH] OR HIV[MeSH] OR hiv[tiab] OR hiv-1*[tiab] OR hiv-2*[tiab] OR hiv1[tiab] OR hiv2[tiab] OR hiv infect*[tiab] OR "human immunodeficiency virus"[tiab] OR "human immunedeficiency virus"[tiab] OR "human immuno-deficiency virus"[tiab] OR "human immune-deficiency virus"[tiab] OR ((human immun*[tiab]) AND (deficiency virus[tiab])) OR AIDS[tiab] OR "acquired immunodeficiency syndrome"[tiab] OR "acquired immunedeficiency syndrome"[tiab] OR "acquired immuno-deficiency syndrome"[tiab] OR "acquired immune-deficiency syndrome"[tiab] OR ((acquired immun*[tiab]) AND (deficiency syndrome[tiab])) OR "sexually transmitted diseases, Viral"[MeSH:NoExp]) AND (performance[tiab] OR results[tiab] OR output[tiab] OR delivery[tiab] OR conditional[tiab] OR contract*[tiab]) AND (financ*[tiab] OR subsid*[tiab] OR remunerat*[tiab] OR pay*[tiab] OR incentive[tiab] OR cash[tiab])

Search strategy for other databases

(hiv OR hiv-1* OR hiv-2* OR hiv1 OR hiv2 OR hiv infect* OR "human immunodeficiency virus" OR "human immunedeficiency virus" OR "human immuno-deficiency virus" OR "human immune-deficiency virus" OR ((human immun*) AND (deficiency virus)) OR AIDS OR "acquired immunodeficiency syndrome" OR "acquired immunedeficiency syndrome" OR "acquired immuno-deficiency syndrome" OR "acquired immune-deficiency syndrome" OR ((acquired immun*) AND (deficiency syndrome)) AND (performance OR results OR output OR delivery OR conditional OR contract*) AND (financ* OR subsid* OR remunerat* OR pay* OR incentive OR cash)
