# Supplementary material for: Performance-based financing for improving HIV/AIDS service delivery: a systematic review
Source: BMC Health Serv Res. 2017 Jan 4;17:6. doi: 10.1186/s12913-016-1962-9 (PMC5210258; doi:10.1186/s12913-016-1962-9)
Supplement: Additional file 2: — Text S2. Effect estimate calculations. (DOCX 23 kb) [file 12913_2016_1962_MOESM2_ESM.docx]

**Text S2.** Effect estimate calculations. Reference: Rothman KJ, Greenland S. Modern epidemiology. 3rd ed. Philadelphia: Lippincott Williams & Wilkins, 2008.

Relative risks and 95% confidence intervals for overall testing, couples testing, and individual not in couple testing for HIV were not reported in: de Walque D, Gertler PJ, Bautista-Arredondo S, Kwan A, Vermeersch C, de Dieu Bizimana J, et al. Using provider performance incentives to increase HIV testing and counseling services in Rwanda. J Health Econ. 2015; **40**: 1-9. However, the data necessary to calculate a relative risk was provided.

**Couples testing**

First, we must calculate the relative risk (RR). We are given β and SE.

RR = e^β

RR = e^0.102 = 1.11

Next, we can calculate the lower and upper limits of the relative risk:


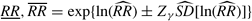
 = e^(β ± 1.96(SE))

= e^(0.102 ± 1.96(0.041)) = 1.02, 1.20

Therefore the relative risk and its 95% confidence interval is: 1.11 (1.02 to 1.20).

**Individual testing**

First, we must calculate the relative risk (RR). We are given β and SE.

RR = e^β

RR = e^0.003 = 1.00

Next, we can calculate the lower and upper limits of the relative risk:


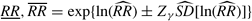
 = e^(β ± 1.96(SE))

= e^(0.003 ± 1.96(0.062)) = 0.89, 1.13

Therefore the relative risk and its 95% confidence interval is: 1.00 (0.89 to 1.13).

Relative risks and 95% confidence intervals for pregnant women testing, pregnant women prophylaxis, and infant prophylaxis were not reported in: Attiah J, Ntumbanzondo M, Ghanotakis E, Katuala G, Pitter C, Buono N, et al. Performance-based financing: a mechanism to improve uptake pediatric and maternal HIV care and treatment. International AIDS Conference; 2010; Vienna, Austria; 2010. However, the data necessary to calculate a relative risk was provided.

**Pregnant women testing**

First, we must calculate the point estimate. We are given A0, N0, A1, and N1:

A0 = 102566 = number of pregnant women receiving testing prior to implementation of PBF

N0 = 141470 = number of pregnant women in antenatal care prior to implementation of PBF

A0/N0 = 0.725

A1 = 10242 = number of pregnant women receiving testing after implementation of PBF

N1 = 10942 = number of pregnant women in antenatal care after implementation of PBF

A1/N1 = 0.936

We can now calculate the point estimate:


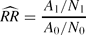


RR = (10242/10942) / (102566/141470) = 1.2911

Next, we must calculate the standard deviation of the log relative risk:


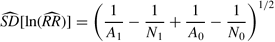


SD[ln(IR)] = (1/10242 - 1/10942 + 1/102566 - 1/141470)^0.5 = 0.00299

Finally, we can calculate the lower and upper limits of the relative risk:


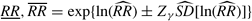


=exp[ln(1.2911) ± 1.96(0.00299)] = 1.2835, 1.2987

Therefore the relative risk and its 95% confidence interval is: 1.29 (1.28 to 1.30).

**HIV positive pregnant women who received ARV prophylaxis**

First, we must calculate the point estimate. We are given A1, A0, and coverage in study arms:

A0 = 3261 = number of pregnant women on prophylaxis prior to implementation of PBF

A0/N0 = 0.614

A1 = 491 = number of pregnant women on prophylaxis after implementation of PBF

A1/N1 = 0.95

Given this information we can calculate N1 and N0:

3261/N0=.614/1

3261/.614=N0

N0=5311= number of pregnant women eligible for prophylaxis prior to implementation of PBF

491/N1 = .95/1

491/.95=N1

N1= 517 = number of pregnant women eligible women eligible for prophylaxis after implementation of PBF

We can now calculate the point estimate:


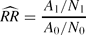


RR = (491/517) / (3261/5311) = 1.547

Next, we must calculate the standard deviation of the log relative risk:


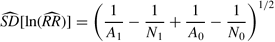


SD[ln(IR)] = (1/491 - 1/517 + 1/3261 - 1/5311)^0.5 = 0.0149

Finally, we can calculate the lower and upper limits of the relative risk:


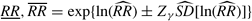


=exp[ln(1.547) ± 1.96(0.0149)] = 1.5025, 1.5928

Therefore the relative risk and its 95% confidence interval is: 1.55 (1.50 to 1.59).

**Infants born to mothers living with HIV who received ARV prophylaxis in delivery**

First, we must calculate the point estimate. We are given A0, N0, A1, and N1:

A0 = 1863 = number of infants receiving prophylaxis in delivery prior to implementation of PBF

N0 = 3931 = number of infants delivered prior to implementation of PBF

A0/N0 = 0.474

A1 = 429 = number of infants receiving prophylaxis in delivery after implementation of PBF

N1 = 471 = number of infants delivered after implementation of PBF

A1/N1 = 0.911

We can now calculate the point estimate:


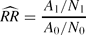


RR = (429/471) / (1863/3931) = 1.922

Next, we must calculate the standard deviation of the log relative risk:


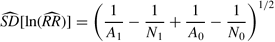


SD[ln(IR)] = (1/429 - 1/471 + 1/1863 - 1/3931)^0.5 = 0.0221

Finally, we can calculate the lower and upper limits of the relative risk:


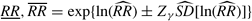


=exp[ln(1.922) ± 1.96(0.022)] = 1.841, 2.007

Therefore the relative risk and its 95% confidence interval is: 1.92 (1.84 to 2.01).

Relative risks and 95% confidence intervals for ART coverage and attrition were not reported in: Tanoh AR, Attiah J, Fayama M, Essombo J, Guebo A. Performance Based Financing: Evaluation of Programatic Results after two years of Implementation in Côte d’Ivoire. 5th IAS Conference on HIV Pathogenesis and Treatment; 2009; Cape Town, South Africa; 2009. However, the data necessary to calculate a relative risk was provided.

**ART coverage**

First, we must calculate the point estimate. We are given A0, N0, A1, and N1:

A0 = 1041 = number of new patients treated with ARV prior to implementation of PBF

N0 = 3720 = number of new patients prior to implementation of PBF

A0/N0 = 0.2798

A1 = 1811 = number of new patients treated with ARV after implementation of PBF

N1 = 3720 = number of new patients after implementation of PBF

A1/N1 = 0.4868

We can now calculate the point estimate:


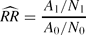


RR = (1811/3720) / (1041/3720) = 1.7397

Next, we must calculate the standard deviation of the log relative risk:


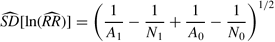


SD[ln(IR)] = (1/1811 - 1/3720 + 1/1041 - 1/3720)^0.5 = 0.0312

Finally, we can calculate the lower and upper limits of the relative risk:


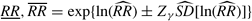


=exp[ln(1.7397) ± 1.96(0.0312)] = 0.7360, 0.9585

Therefore the relative risk and its 95% confidence interval is: 1.74 (1.64 to 1.85).

**Attrition at 12 months**

First, we must calculate the point estimate. We are given N1, N0, and the attrition rate in each of the study arms:

N0 = 1041 = number of patients prior to implementation of PBF

A0/N0 = 0.2667

N1 = 1811 = number of patients after implementation of PBF

A1/N1 = 0.224

Given this information we can calculate A1 and A0:

A0/1041=0.2667/1

A0=0.2667*1041

A0 = 278 = number of patients lost-to-follow up prior to implementation of PBF

A1/1811=0.224/1

A1=0.224*1811

A1 = 406 = number of patients lost-to-follow up after implementation of PBF

We can now calculate the point estimate:


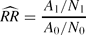


RR = (406/1811) / (278/1041) = 0.8399

Next, we must calculate the standard deviation of the log relative risk:


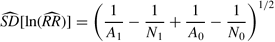


SD[ln(IR)] = (1/406 - 1/1811 + 1/278 - 1/1041)^0.5 = 0.0674

Finally, we can calculate the lower and upper limits of the relative risk:


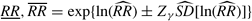


=exp[ln(0.8399) ± 1.96(0.0674)] = 0.7360, 0.9585

Therefore the relative risk and its 95% confidence interval is: 0.84 (0.74 to 0.96).
