# Supplementary material for: Performance-based financing for improving HIV/AIDS service delivery: a systematic review
Source: BMC Health Serv Res. 2017 Jan 4;17:6. doi: 10.1186/s12913-016-1962-9 (PMC5210258; doi:10.1186/s12913-016-1962-9)
Supplement: Additional file 3: Table S1. — Bias assessment of randomised trials. (DOCX 12 kb) [file 12913_2016_1962_MOESM3_ESM.docx]

**Table S1**. Bias assessment of randomised trials. + indicates Yes, - indicates No

|  |  | **Selection bias** | |  | **Performance and detection bias** |  | **Attrition bias** |  | **Reporting bias** |  |  |
| --- | --- | --- | --- | --- | --- | --- | --- | --- | --- | --- | --- |
| Author |  | Adequate sequence generation | Allocation concealment |  | Blinding of participants, personnel and outcome assessors |  | Incomplete outcome data addressed |  | Free of selective reporting |  | Free of other bias |
| De Walque |  | - | - |  | - |  | + |  | + |  | + |
