# Supplementary material for: Performance-based financing for improving HIV/AIDS service delivery: a systematic review
Source: BMC Health Serv Res. 2017 Jan 4;17:6. doi: 10.1186/s12913-016-1962-9 (PMC5210258; doi:10.1186/s12913-016-1962-9)
Supplement: Additional file 4: Table S2. — Bias assessment of observational studies. (DOCX 12 kb) [file 12913_2016_1962_MOESM4_ESM.docx]

**Table S2**. Bias assessment of observational studies. + indicates Yes, - indicates No

|  | **Selection bias** | | |  | **Confounding** |  | **Measurement bias** | | |
| --- | --- | --- | --- | --- | --- | --- | --- | --- | --- |
| Author | Representativeness of the health facilities providing PBF to the average health facility in the country. | Representativeness of the health facilities not providing PBF to the facilities providing PBF. | PBF ascertained in health facilities. |  | Demographic variables included in analytical model |  | Outcomes were validated. | Median or mean follow-up of at least six months. | ≤ 20% of participants were lost to follow-up |
| Attiah | + | + | + |  | - |  | + | + | + |
| Odeny | + | + | - |  | + |  | + | + | - |
| Tanoh | + | + | + |  | - |  | + | + | - |
